# Early Diagnosis and Intervention for Autism Spectrum Disorder

**DOI:** 10.7759/cureus.89591

**Published:** 2025-08-07

**Authors:** Paula Vanegas Navarro, Fiorella Apuy Rodríguez, María Jesús Arias Alvarado, Melissa Chacón Quirós

**Affiliations:** 1 Faculty of Medicine, Universidad de Costa Rica, San José, CRI

**Keywords:** autism spectrum disorder, behavioral interventions, developmental surveillance, diagnostic criteria, neurodevelopment

## Abstract

Autism spectrum disorder (ASD) is a neurodevelopmental syndrome that impacts two main areas: social communication and restrictive or repetitive behaviors. Other symptoms and comorbidities may be manifested, according to the different clinical presentations and severity levels. ASD diagnosis can be performed by two years of age; however, certain diagnostic challenges may lead to a late diagnosis and significant intervention delay. Studies have shown that early diagnosis and interventions have a positive impact on the child’s development, acquisition of social or language skills, and overall quality of life. This review aims to explore the appropriate implementation of standardized, efficient diagnostic methods and criteria at primary care routine consultations, and prompt interventions that must follow in high-risk children.

## Introduction and background

Autism spectrum disorder (ASD) is a neurodevelopmental syndrome that encompasses two main areas of impairment: social communication and restrictive or repetitive behavior. It presents a heterogeneous phenotype and polygenic heritability, resulting in various clinical presentations and severity levels [1]. Its etiology hasn’t been determined; however, studies have pointed toward interaction between genetic and environmental elements (such as prenatal or perinatal complications) through several mechanisms such as oxidative stress, inflammation, and endocrine disturbances [1-2].

The prevalence of this entity has recently increased, currently estimated to be 0.5-1% worldwide [3]. This is largely due to less restrictive diagnostic criteria and better diagnostic processes. Other factors, such as a surge in community awareness and parents’ quick identification of red flags in their children, have contributed to the early diagnosis [2-3]. This entity’s prevalence ratio is 4:1 males vs females, probably due to neurobiological and hormonal factors [4]. Besides the evident consequences of this entity’s rising prevalence in the medical field, additional aspects must be taken into account, such as its educational, social, and economic repercussions [5].

Given its high prevalence and significant repercussions, certain clinical and diagnostic challenges must be addressed. Theoretically, ASD can be diagnosed between 12 and 24 months of age, and severe cases may be identified in children with red flags by 12 months old [1]. However, in clinical practice, the age of diagnosis is usually between four and five years old, thus delaying prompt interventions which could improve long-term outcome [2]. This diagnostic lag may be due to socioeconomic disparities, inefficient diagnostic methods, difficulty identifying the wide variety of symptoms or severity levels, and symptom onset at different ages [4]. Given its rising prevalence and impact on children’s development, this issue has become a public health concern. Several strategies have been applied in order to shorten the diagnosis-intervention delay, such as efficient diagnostic tools, medical consultation, referral, and early interventions [3, 6].

## Review

Methodology

For this narrative review, databases such as EBSCO, PubMed, and Elsevier were searched for articles published from 2016 to 2024. The following keywords were researched: “autism spectrum disorder”, “epidemiology”, “early diagnosis”, “early intervention”, “prognosis”, “risk factors”, “etiology”, “diagnostic criteria”, and “DSM-5”. After performing the initial selection, articles that complied with the following were included: studies published within the last ten years, review articles, and meta-analyses concerning early diagnosis and intervention in ASD.

Risk factors and red flags

First of all, within routine clinical evaluations, it remains important to notice risk factors or red flags for ASD, which may be displayed early on. Major risk factors include positive family history (7-19% recurrence risk in siblings) and male sex. Other risk factors include low birth weight, prematurity, and birth complications. Some red flags, which typically manifest in high-risk children during the first year of age, are: delayed motor control, poor visual tracking and face processing, sleeping and feeding struggles, excessive passivity or reactivity, and lack of joint-attention development by 15-18 months of age [2, 6-7].

Clinical presentation and diagnostic criteria

As children with ASD grow up, they may display behaviors that fall within two main symptom categories: social interaction impairment and restricted or repetitive behaviors and interests. These behaviors are usually manifested in children between 18 and 24 months of age, and are typically raised as concerns, which parents consult the child’s pediatrician or primary care physician [2, 8].

According to the DSM-5 criteria, in order to establish a diagnosis of ASD, the patient must exhibit the following behaviors in social interaction: inability to show emotional reciprocity, impairment of non-verbal communication, and difficulty engaging and maintaining relationships [2]. Early representations of these behaviors include children who don’t exhibit a social smile or adopt the anticipatory posture before being picked up. During these early years, predictors of future development of ASD might include: maintaining poor eye contact, exhibiting a poor response when their name is called, poor joint attention (use of gaze and gestures in order to share captivating objects with other people), absence of pointing, and atypical attachment behaviors, such as not acknowledging significant people in their lives or presenting less stranger-related anxiety compared to other children [1, 6]. Higher functioning children can mask their social deficits as they grow older; however, certain behaviors may still identify their impairments, for example, less engagement in spontaneous play or conversation, few shared interests with other children, and few non-verbal gestures during conversations. They also encounter difficulties when inferring other people’s feelings, emotional state, or intentions [1].

In the domain of restrictive and repetitive behaviors, patients manifest at least two out of the following four sub-criteria: stereotyped behaviors and language, insistence on routine, fixed interests, and unusual sensitivity to sensory stimuli [2]. Young children may exhibit restricted exploratory play, using toys less symbolically and more ritualistically. They also lack the same level of imitative or abstract play as their peers. These children prefer to engage in repetitive activities, such as spinning or lining up items, and present strong attachments to certain objects. Additionally, they maintain a profound insistence on routine, and experience severe anxiety or tantrums when it’s disrupted [1].

There are further behavioral symptoms, not included in the previous categories, that are typically present in children with ASD. Some may experience impairment in language development, and in severe cases, may trigger a language delay (up to 50% of them may never develop functional speech). In less severe cases, they display minimal babbling during the first year of age, stereotyped noises or syllables, echolalia, or carry on conversations without typical inflections. Psychiatric comorbidities or emotional disturbances, such as irritability, self-injury, unstable mood, inattention, hyperactivity, and insomnia, may also be present [1].

Assessment tools

Although the DSM-5 has established diagnostic criteria for ASD, clinical diagnosis according to these indicators still has certain limitations, given that they are not considered a proper diagnostic test for ASD. In response to this issue, other tools have been developed in order to reach an accurate diagnosis for ASD, for example: parental questionnaires and interviews, clinical assessment, and personal interactions with the child [5, 9].

One of the most important diagnostic methods is the developmental surveillance performed at scheduled health visits. Physicians record information provided by caregivers regarding the achievement of developmental milestones, by listening to parents’ concerns, carrying out a clinical observation of the child, and incorporating standardized measures, such as parent questionnaires. This assessment is particularly important at the 18-month-old consultation, given that at this age, typical ASD symptoms can appear [2].

Standardized measures are performed on children with an increased risk for ASD, which may detect ASD earlier and consistently. These methods are chosen according to the format desired, based on parents’ report or direct child assessment, according to the skill wished to be measured, and the target population. Some of the most popular screening tools are: the Modified Checklist for Autism in Toddlers, Revised with Follow-Up (M-CHAT-R/F), which holds high specificity (96%) and sensitivity (91%), and can be performed between 16-30 months old, the Ages and Stages Questionnaire (ASQ), the Screening Tool for Autism in Toddlers (STAT), and Social Communication Questionnaire (SCQ) [5, 9]. Another early screening tool is the Social Attention and Communication System (SACS), which can identify red flags in high-risk children at 8, 12, 18, and 24 months of age [6].

Other important tests for diagnosing ASD are the Autism Diagnostic Observation Schedule (ADOS) and the Autism Diagnostic Interview-Revised, which include clinical and questionnaire-type assessment tools. In the ADOS, the medic engages in a structured play with the child in order to assess their social skills. On the other hand, the Autism Diagnostic Interview encompasses a questionnaire, answered by the caregiver, about the child’s behavioral and developmental symptoms [5].

Diagnostic limitations

Despite the DSM-5 diagnostic criteria and multiple “gold-standard” diagnostic tools, certain challenges persist in the achievement of an early diagnosis. Factors concerning the patient’s context include socioeconomic, geographical, or cultural inequities in access to medical care and a lack of awareness of red flags in parents or medical professionals [10-11]. Even the implementation of “gold-standard” diagnostic tools may display some challenges, such as a bias from the caregiver’s memory or understanding when remembering the child’s symptoms, and external factors that may affect the doctor-patient interaction, modifying the test’s ability to produce a reproducible, accurate diagnosis. These factors might influence the application of a “standardized” diagnostic assessment [5]. Additionally, limitations when performing a clinical diagnosis include (1) diagnostic symptoms that don’t manifest until the first year of age (thus establishing a lower age limit for diagnosis), (2) a wide range of symptom manifestation and severity, (3) some behaviors being considered normal during early childhood (they only become pathological if they persist beyond that age), and (4) certain diagnostic behaviors (for example, repetitive mannerisms) which may appear in older children [5, 9].

Besides the restrictions mentioned for clinical diagnosis, recent studies have also shown limitations in the use of certain questionnaires, such as the M-CHAT-R/F. The use of this test as a universal screening tool has recently been studied, and it has been concluded that, given its high specificity-sensitivity level and the general population’s low prevalence rate (2%), this tool provides a higher percentage of false-positive results. Thus, it has been estimated to have a 33% accuracy rate when applied to children universally, without parents raising concerns about their behavior [5].

Therefore, ASD diagnosis cannot be based solely on a single universal diagnostic tool. Instead, different steps for test applications, issued by a multidisciplinary team, have been established in order to arrive at more accurate diagnoses. As mentioned above, normal developmental surveillance must be performed at every well-child consultation, and parents’ behavioral concerns about their child must be addressed and further explored. According to the initial evaluation’s findings, a diagnostic tool, such as the M-CHAT-R/F, may be used (this particular questionnaire is used in children under 30 months old). After performing the first screening test, or “Level 1”, the subject pool ASD prevalence significantly rises from 2% (general population prevalence rate) to approximately 32%. Afterwards, the patients are referred to an appropriate specialist and can undergo “Level 2” tests, for example, the Rapid Interactive Screening Test for Autism in Toddlers (RITA-T), where the physician performs a confrontational interactive assessment of the child. Finally, children who display altered results in these tests must undergo an evaluation through gold standard tools, such as the ADOS. After the diagnosis has been established, the physician must identify the child’s functional limitations in order to elaborate a report and justify the need for specific, prompt interventions [5, 7].

An important factor that must be taken into account during this diagnostic process is the information provided to the parents. Given the prolonged diagnostic assessment, parents may experience high stress levels due to not being provided direct updates. In order to alleviate the parents’ worries, the physician must issue relevant information during each diagnostic step, provide education about the ASD diagnosis and its implications, and overall support, considering the family’s social and ethnic backgrounds [7].

Given these difficulties in accurately determining an early diagnosis, current research is aiming to develop objective biomarkers (such as neuroimaging and epigenetic variations) in order to standardize early ASD diagnosis, used in combination with screening tools [8]. Some of the biomarkers currently studied are: mRNA expression profiles, maternal and newborn immunoglobulin levels, EEG and MRI imaging (which may show atypical cortical activation, brain development trajectories, and functional connectivity). Other particular biomarkers being developed are gaze metrics, where abnormal visual orienting can be objectively identified. [7] These biomarkers may provide certain benefits and further efficiency in early diagnosis, such as determining the child’s risk in order to be screened, their use as a secondary screening tool, and as a confirmation of the behavioral assessments or stratification of symptom severity [5, 7].

Prognosis

As mentioned above, ASD involves a heterogeneity of clinical presentations and severity levels, which impact each child differently. Children with IQs over 70, with standard adaptive skills and acquisition of language by five or seven years of age, show better prognoses. Studies have shown that early behavioral interventions lead to a positive impact in the child’s development, preventing symptom progression and allowing them to function within the average range according to their age group. This encompasses an improvement in communication, social interaction, adaptive behaviors, and cognitive levels. Children with repetitive behaviors can also benefit, thus reducing their persistent conduct [1, 3, 10-12].

Early diagnosis leading to prompt interventions (before two years old) is of particular importance, given that the brain presents a greater level of neuroplasticity during infancy and potential for modification in abnormal reward circuits. At this age, interventions are more effective in providing better social or communication skills and quality of life during adulthood. This impact also extends to the patient’s family, reducing emotional and financial stress levels [4, 6, 12-14].

Early interventions

As soon as the first-contact physician suspects ASD or identifies a high-risk child, they must begin the appropriate therapies [13]. These might include psychosocial interventions, speech therapy, and behavioral or pharmacological treatment for irritability, according to the child’s clinical presentation [1, 12].

Early intensive behavioral interventions (EIBI) and applied behavior analysis (ABA) are some of the most widely studied therapies. These are provided in weekly consultations, where social, language, or play skills are addressed [10]. The Early Start Denver Model targets play and relationship skills in young children, within their daily routine (at home or daycare), aided by parents as co-therapists. These early interventions greatly benefit from being transferred to the child’s everyday life, giving special importance to family involvement and support [6]. Parents’ attitudes or levels of stress toward these interventions have been proven to have repercussions on the impact of these therapies on the children. A study in the United Kingdom showed that interventions in which parents reported lower stress levels were associated with better intellectual and educational outcomes in their children. The opposite was shown in children whose parents reported higher stress levels [15]. On the other hand, social skills training typically takes place in a group setting, where children initiate conversations or games and practice emotional identification. Studies have shown that cognitive-behavioral therapy is effective for anxiety, depression, or obsessive-compulsive comorbidities. Additionally, in school settings, certain educational adjustments may be applied, such as incorporating visual aids or computer programs in order to facilitate the learning process [1].

Finally, early behavioral therapy can be used, in combination with pharmacological treatment, in order to reduce repetitive, distressing, or self-harming behaviors [8]. In the case of irritability, two second-generation antipsychotics, risperidone (typical first-line treatment in children between five and 16 years old) and aripiprazole, have been approved by the FDA. Risperidone has also shown high levels of efficacy in aggressive children and those with self-harming, repetitive, or restrictive behaviors. Certain alternative treatments may be used in combination with pharmacological and behavioral interventions, such as music therapy, yoga, melatonin, and multivitamins. These practices are considered safe; however, their particular efficacy is still unknown [1].

## Conclusions

In today’s settings, ASD has presented an increase in prevalence, becoming a public health concern. Multiple studies have shown that early diagnosis and interventions lead to a better prognosis, acquisition of social or language skills, and improved overall quality of life for the patient and their families. However, in clinical settings, certain limitations arise in the achievement of an early diagnosis and intervention, such as variable clinical presentations or age of manifestation, socioeconomic or cultural settings, and diagnostic methods with low accuracy or efficiency.

Certain considerations in the implementation and choice of diagnostic tools have been taken into account in order to efficiently identify children with ASD. The preferred diagnostic process, from Level 1 questionnaires up to Level 2 and clinical assessments, has proven to be a more coherent and structured method in order to achieve ASD detection. Therefore, accurate diagnostic methods and referral to the appropriate professionals must be reinforced in primary care physicians and pediatricians in order to reduce the diagnostic-intervention delay.

Once early diagnosis and treatment have begun, children may benefit from a significant improvement in communication, behavioral, and cognitive skills, thus improving their future development and functionality.
